# Optimizing Stereotactic Intracranial Neoplasm Treatment: A Systematic Review of PET Integration with Gamma Knife Radiosurgery

**DOI:** 10.3390/diseases13070215

**Published:** 2025-07-10

**Authors:** Robert C. Subtirelu, Eric M. Teichner, Milo Writer, Kevin Bryan, Shiv Patil, Talha Khan, Lancelot Herpin, Raj N. Patel, Emily Christner, Chitra Parikh, Thomas Werner, Abass Alavi, Mona-Elisabeth Revheim

**Affiliations:** 1 Department of Radiology, Hospital of the University of Pennsylvania, Philadelphia, PA 19104, USAkevbryan@sas.upenn.edu (K.B.); talha_khan@med.unc.edu (T.K.); rajnpatel05@gmail.com (R.N.P.); ekc64@drexel.edu (E.C.);; 2 Sidney Kimmel Medical College, Thomas Jefferson University, Philadelphia, PA 19107, USA; 3 The Intervention Centre, Division of Technology and Innovation, Oslo University Hospital, 0372 Oslo, Norway; 4 Institute of Clinical Medicine, University of Oslo, 0313 Oslo, Norway

**Keywords:** intracranial neoplasms, gamma knife radiosurgery (GKRS), positron emission tomography (PET), radiotracers (^18^F-FDG, ^11^C-MET, ^18^F-FET, and ^18^F-DOPA, ^18^F-FAZA, ^68^Ga-DOTATOC, FRP-170), tumor delineation, post-operative monitoring, combination imaging (PET/CT, PET/MR), microscopic infiltrations, radiation necrosis, lesion recurrence, metabolic tumor volume (MTV), blood–brain barrier (BBB), radiotracer uptake, neuro-oncology, radiosurgical outcomes

## Abstract

Objective: Traditional imaging modalities for the planning of Gamma Knife radiosurgery (GKRS) are non-specific and do not accurately delineate intracranial neoplasms. This study aimed to evaluate the utility of positron emission tomography (PET) for the planning of GKRS for intracranial neoplasms (ICNs) and the post-GKRS applications of PET for patient care. Methods: PubMed, Scopus, and ScienceDirect were searched in order to assemble relevant studies regarding the uses of PET in conjunction with GKRS for ICN treatment. PRISMA (Preferred Reporting Items for Systematic Reviews and Meta-Analyses) guidelines were followed to identify relevant studies on the use of PET in conjunction with GKRS. Particular emphasis was placed on review articles and medical research investigating tumor delineation and post-operative care. Relevant studies were selected and assessed based on quality measures, including study design, sample size, and significance. Inclusion and exclusion criteria were used to examine the yield of the initial search (n = 105). After a secondary review, the included results were identified (n = 50). Results: This study revealed that PET imaging is highly accurate for the planning of GKRS. In fact, many cases indicate that it is more specific than traditional imaging modalities. PET is also capable of complementing traditional imaging techniques through combination imaging. This showed significant efficacy for the planning of GKRS for ICNs. Conclusions: While PET shows a multitude of applications for the treatment of ICNs with GKRS, further research is necessary to assemble a complete set of clinical guidelines for treatment specifications. Importantly, future studies need a greater standardization of methods and expanded trials with a multitude of radiotracers.

## 1. Introduction

Stereotactic radiosurgery uses focused beams of radiation to damage the DNA of a targeted tumor cell without incisions. First proposed by Dr. Lars Leksell for the noninvasive targeting of brain portions for irradiation [1], this is achieved through the guiding of gamma radiation emitted by the cobalt-60 isotope from multiple beams to target specific masses and minimize radiation injury to the surrounding tissue. The Gamma Knife technology generates a three-dimensional model of the patient’s brain, allowing clinicians to prescribe specific doses of radiation. The intention of this method was for these doses to become insignificant outside of the targeted tumor volume. While many imaging modalities have been used to guide Gamma Knife, this review will focus on positron emission tomography for the metabolic mapping of intracranial neoplasms (ICNs).

Positron emission tomography (PET) is a highly specific imaging modality for the visualization of molecular pathways within the human body [2]. This is in part due to the plethora of available radionuclides that can be used in conjunction with PET. Typically, the significant differences between radiotracers relate to half-life and the pathway of metabolization, which can impact opportunities for application. In the last 60 years, PET technology has advanced in both spatial resolution and sensitivity [3], increasing the applications of this technology.

Neoplasms are abnormal growths of tissue that can occur in various parts of the body, including the central nervous system. Brain neoplasms, which include both benign and malignant tumors, require careful treatment planning. Current methods of treatment rely on initial surgical resection of neoplastic tissue to maximize removal. This is followed by supplementary chemotherapy and radiotherapy [4]. The identification and monitoring of tumor growth and infiltration are necessary to ensure patient survival and to prevent tumor recurrence [5,6]. Stereotactic radiosurgery (SRS) has been shown to be an effective treatment for brain tumors [7], although patient success post-treatment is reliant on the imaging modalities used during the planning process and treatment fractionation. Hypofractionated treatment has proven effective for achieving local tumor control [8,9]. The effectiveness of radiotracer-guided positron emission tomography (PET) for the planning of Gamma Knife radiosurgery (GKRS) in the treatment of neoplasms as the main imaging protocol, rather than supplementary, is controversial [10,11,12,13,14]. This is due to the need for radiotracer selection on a case-by-case basis to accomplish clinical goals [15]. This is significant due to radiotracer differences lying in various paths of metabolization. In the case of 2-[^18^F]-fluoro-2-deoxy-D-glucose ([^18^F]FDG)-PET, the metabolization of the tracer relies on glucose transporters and mimics the pathway of glucose before being suspended in the cytoplasm after phosphorylation to FDG-6-phosphate [16].

Chemotherapy as a supplementary treatment in conjunction with GKRS shows promise for contributing to long-term patient survival [17].

The use of PET in conjunction with other imaging modalities can supplement the metabolic data collected by PET with anatomical data in the case of supplementary Magnetic Resonance Imaging (MRI) [6]. This discipline, known as radiomics, relies on the idea that nuanced changes in tumor structure can be observed with the use of multiple imaging protocols simultaneously [18]. This technique is especially useful for distinguishing between tumor recurrence and radiation necrosis.

While maximal cancerous tissue removal is a priority for treatment, accurate tumor delineation is just as significant to patient success [19]. The use of Stereotactic Radiosurgery (SRS) in tumor treatment results in 26% of patients developing radiation injury [7]. This is often caused by insensitive imaging software that obscures tumor borders and results in the irradiation of the surrounding tissue. Typical modalities of neoplasm detection in preparation for surgery and in post-operative patient monitoring may confuse necrotic tissue with tumor recurrence [20]. This can lead to unnecessary and damaging post-operative care for a patient, including additional rounds of radiosurgery.

This review aims to summarize the literature on [^18^F]FDG, [^11^C]methionine ([^11^C]MET), [^18^F]fluoro-ethyl-tyrosine ([^18^F]FET), 6-[^18^F]fluoro-l-dopa (^18^F]FDOPA), and other radiotracers in conjunction with PET for the planning of stereotactic radiosurgery for neoplasms. Furthermore, the use of PET/MR and PET/Computed Tomography (CT) combination imaging is analyzed for its effectiveness in tumor delineation, where radiotracer-guided PET may fall short in accuracy and sensitivity [21]. The most significant point of controversy among physicians regarding the use of PET for the planning of GKRS is whether it is adequate to act as the principal imaging protocol. Differences in the metabolization, half-life, and metabolic behavior of radiotracers tend to be responsible for these claims. This review distinguishes the nuances between [^18^F]FDG, [^11^C]MET, [^18^F]FET, and [^18^F]FDOPA in order to determine the ideal circumstances of use for each tracer. A greater understanding of the specific advantages of each radiotracer would allow for the expansion of GKRS as a treatment for ICNs with an ensured increase in accuracy, sensitivity, and specificity from traditional methods.

## 2. Methods

### 2.1. Literature Search and Study Selection

**Figure 1:**
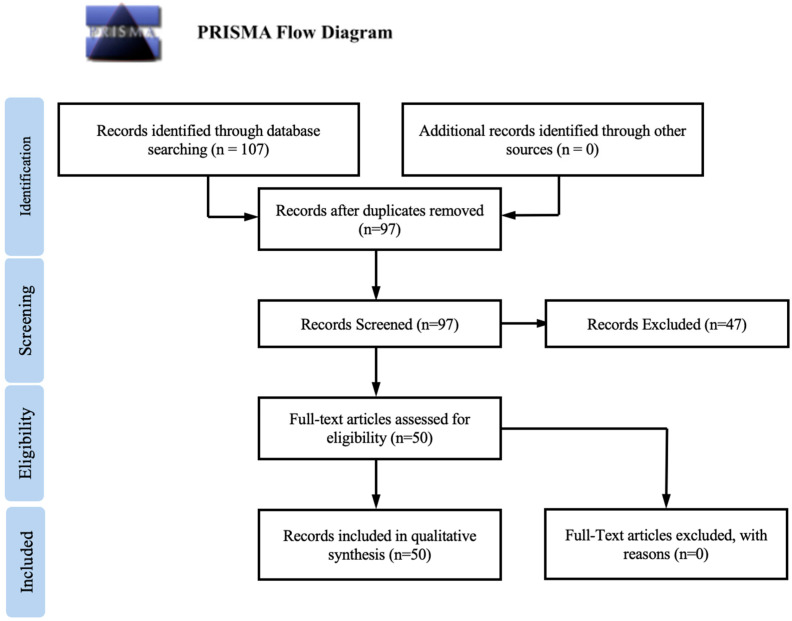
PRISMA flow diagram.

To conduct an extensive literature search, we developed a comprehensive search strategy and performed searches on PubMed, SCOPUS, and ScienceDirect databases spanning from 1995 to 2025. Database-specific logic and wildcards were applied. Supplemental hand searching was performed to identify additional records. Our searches yielded 107 results. We followed the PRISMA guidelines to identify relevant studies. The protocol has not been registered. Two independent reviewers evaluated the titles and abstracts of the gathered publications, and any discrepancies were resolved by a third reviewer who carefully assessed the full texts of the remaining studies. After exclusion of duplicates, 97 results remained for screening. Following the screening process, 47 articles were removed in accordance with the set inclusion and exclusion criteria, leaving 50 articles suitable for review. A PRISMA flow diagram illustrating this selection process is presented in Figure 1.

Articles were evaluated based on overall quality, study design, relevance to the research question, and the clarity and consistency of reported patient outcomes. Inclusion criteria required that studies explicitly assess the use of positron emission tomography (PET) imaging in the context of intracranial neoplasm (ICN) management, specifically focusing on its role in target delineation for Gamma Knife radiosurgery (GKRS) planning and/or the evaluation of post-treatment outcomes such as tumor recurrence, progression, or radiation-induced changes. Eligible studies included clinical trials, cohort studies, and case series with clear descriptions of patient selection, imaging modalities used, radiotracers administered, and outcomes measured. Studies were included regardless of tumor origin (primary/metastatic) or histology. Studies were excluded if they involved non-human subjects, were non-clinical or preclinical in nature, or lacked sufficient methodological detail to permit critical appraisal. Additional exclusions were applied to letters to the editor, editorials, and conference abstracts. Studies with incomplete data reporting or those not available in full-text were also omitted from the final analysis.

### 2.2. Data Extraction and Quality Assessment

Two independent reviewers performed a data extraction from the 50 eligible articles. A third reviewer was consulted and resolved any discrepancies. The following information was extracted from each included study: study title, author, year of publication, sample size, imaging modality, PET radiotracer used, and reported outcomes, including focal recurrence and radiation necrosis.

### 2.3. Data Analysis

A descriptive analysis was executed to summarize the circumstances and findings of the outlined studies. The described outcomes, such as decreased focal recurrence and differing degrees of radiotracer accuracy, were synthesized and presented. A synthesis of the findings of the included studies was discussed in the context of the objectives of this review, mainly the improvement of relevant clinical outcomes. The limitations of the current clinical knowledge and potential sources of bias within the examined studies were also evaluated during data analysis.

## 3. Results

A total of 107 studies across PubMed, SCOPUS, and ScienceDirect databases from 1995 to 2025 were initially screened. Following the removal of duplicate articles and further elimination based on set inclusion and exclusion criteria, 50 articles remained that were appropriate for review.

The selected articles referred to various PET imaging modalities and radiotracers for the planning of GKRS, including [^18^F]FDG, [^11^C]MET, [^18^F]FET, [^18^F]FDOPA, and [^68^Ga]Ga- DOTA-D-Phe-Tyr3-octreotide ([^68^Ga]Ga-DOTATOC). Most studies were observational or retrospective in nature, with several randomized controlled trials (RCTs). Sample sizes for these studies ranged from tens to hundreds of patients. A summary of the literature is provided in Table 1.

### 3.1. Planning of Radiosurgery

The specificity and sensitivity of PET imaging to provide metabolic data can supplement routine MRI in the planning of GKRS. The integration of PET in several radiosurgery studies was found to markedly improve tumor delineation and optimize target selection. Additionally, PET can aid tumor management during surgical and post-operative periods.

### 3.2. Radiotracers

While [^18^F]FDG has emerged as the dominant radiotracer for PET imaging, this modality is limited by low tumor clarity due to high brain uptake. [^18^F]FDG is still highly valuable, however, as this approach can more accurately differentiate between malignant and benign meningiomas than other tracers such as 1-11C-acetate ([^11^C]acetate). Amino acid tracers such as [^11^C]MET, [^18^F]FET, and [^18^F]FDOPA benefit from high tumor-to-background ratios that can improve tumor delineation. Of particular interest is [^18^F]FET given its ability to provide dynamic data, resistance to metabolic degradation, and viability in the imaging of cerebral neoplasms. Somatostatin receptor subtype 2 (SSTR-2)-based PET modalities, including [^68^Ga]Ga-DOTATOC, [^68^Ga-DOTA,Tyr3]-octreotate ([^68^Ga]Ga-DOTATATE), and [^68^Ga]Ga-DOTA-1-

NaI3-octreotide ([^68^Ga]Ga-DOTANOC), demonstrate diagnostic utility specifically in meningiomas. In the pre-operative monitoring of hypoxic lesions, [^18^F]fluoro-methyl- 2- nitroimidazole ([^18^F]FRP-170) and [^18^F]fluoroazomycin arabinoside ([^18^F]FAZA) have been explored for correlations with immunohistochemical biomarkers.

### 3.3. Differentiating Between Lesion Recurrence and Radiation Necrosis

PET is capable of visualizing lesions with high metabolic activity, thereby providing a means to distinguish stable lesions from those likely to progress. In doing so, PET reduces unnecessary radiation exposure to non-cancerous tissue and the likelihood of radiation necrosis. Treatment guided by structural imaging modalities such as MRI alone has been linked to poorer survival outcomes compared to PET-guided treatments. [^18^F]FDG PET, however, falls short of the desired accuracy provided by histopathology in differentiating between tumor cells and necrotic tissue. [^11^C]MET PET was found to have a high diagnostic accuracy in this differentiation, though the specificity of this modality is still insufficient for the actual planning of GKRS.

### 3.4. Combination Imaging

Supplementing anatomical information from MRI or CT imaging with PET-acquired metabolic data is routinely conducted both pre- and post-operatively. While the low spatial resolution of PET alone can challenge its application in clinical settings, combination imaging has demonstrated the utility of PET in tumor detection and management.

### 3.5. Microscopic Infiltrations

The microscopic infiltrations of ICNs are a critical target for radiosurgical elimination due to their correlation with tumor recurrence. The identification of these microscopic extensions with MRI or CT is an ongoing challenge. Similar to MRI, [^18^F]FDG PET suffers from a low tumor-to-background ratio that limits its application in this context. [^68^Ga]Ga-DOTATOC PET/CT, however, has shown great potential in delineating microscopic tumor tissue that is often missed in other imaging modalities.

## 4. Discussion

### 4.1. Planning of Radiosurgery

While MRI has remained the routine imaging modality for the planning of GKRS, PET has emerged as a highly accurate alternative with improvements in target selection and tumor specificity. PET measures the tissue uptake of radiolabeled tracers, which emit positrons that merge with electrons to create measurable gamma rays [14]. Importantly, the function of PET relies on the ability of a radiotracer to be uptaken in specific tissues. Research is still necessary to understand the ideal clinical applications of these technologies; however, their uses are promising for the field of neuro-oncology.

GKRS requires imaging of utmost accuracy because a single dose session lacks spatial control other than the imaging with which it is provided. In a study performed by Levivier et al., PET/MR was used to define 102 target volumes. Of these, abnormal PET uptake was discovered in 86%. This information significantly changed the definition of 73% of the MR-defined tumors [49].

PET was confirmed to provide additional information to previous imaging modalities, which can aid in the planning of GKRS. Schindler et al. noted that a patient treated with GKRS for a pituitary macroadenoma was diagnosed with complex partial seizures 15 months post-surgery [31].

Levivier et al. incorporated fiducial marker imaging to use PET data in association with GammaPlan. Researchers used a phantom to confirm that PET data allow for high spatial accuracy [22]. Furthermore, PET/MR imaging enabled the definition of target volume in gliomas. This is especially beneficial in cases with poorly defined lesions and microscopic infiltrations. The use of PET data in the planning of GKRS allows for the use of metabolic data in conjunction with anatomical data gathered from MR imaging. Not only does this improve radiosurgical outcomes, but it also allows for improved monitoring of lesion metabolism post-surgery.

PET imaging improves the handling of pediatric brain tumors during the planning, surgical, and post-surgery stages. The management of incidentally identified tumors, as well as tumors that were poorly defined using MRI, was greatly improved by the use of PET imaging. Furthermore, the detection of evolving tissue and differentiating it from indolent structures was aided by PET. Typically, MRI struggles to optimize target selection for the planning of radiosurgery for ICNs. PET does not have this problem. A study conducted by Pirotte et al. showed that the functional data introduced by PET during the planning of radiosurgery can greatly contribute to the effectiveness of GKRS in pediatric ICNs [12].

### 4.2. Radiotracers

George Charles de Hevesy is credited with identifying the Radioactive Tracer Theory, the basis of nuclear medicine. Radiotracers used in conjunction with positron emission tomography consist of a positron-emitting isotope that is bound to an organic ligand. The isotope tag allows the PET machine to detect the presence and relative concentration of the tracer. The ligand can be chosen to target a specific protein. This is the cause of the differing behaviors of tracers. The major radiotracers focused on in this review are [^18^F]FDG, [^11^C]MET, [^18^F]FET, FDOPA, and DOTATOC. [^18^F]FDG has been mainly used clinically for whole-body cancer staging and different brain pathologies. [^18^F]FDG relies on elevated glucose metabolism in tissues in order to accumulate in clinically relevant tissues, such as cancer. Due to the normally increased glucose metabolism of the brain parenchyma, [^18^F]FDG has a low signal-to-noise ratio for brain tumors [15]. The sensitivity of [^18^F]FDG has been shown to be lower than that of amino acid tracers. [^18^F]FDG has 84% sensitivity in high-grade glioma patients, whereas [^11^C]MET has a 93% sensitivity [54]. Amino acid tracers have become standard for brain tumor imaging. Of this class of radiotracers, [^11^C]MET has been the most characterized. All three amino acid radiotracers accumulate in gliomas and brain metastases due to increased transport by LAT1 and LAT2 [15]. Clinically, [^18^F]FET has logistical advantages compared to other amino acid tracers. [^18^F]FET has a half-life of 110 min, whereas MET has a half-life of 20 min. The short half-life of MET requires centers to have an onsite cyclotron unit. PET imaging of brain tumors using MET and FET have similar results [15]. Lastly, [^68^Ga]Ga-DOTATOC is commonly used in clinical settings for detecting neuroendocrine tumors. [^68^Ga]Ga-DOTATOC targets somatostatin receptors, more specifically, SSTR2 and SSTR5. Neuroendocrine tumors overexpress different SSTR subtypes depending on the origin and grade of the tumor [55].

[^18^F]FDG has arisen as the dominant radiotracer for PET, mainly because of its efficient metabolization. The expression of Glucose Transporter 1 (GLUT-1) by the blood–brain barrier (BBB) and glial cells allows for the ease of transport of [^18^F]FDG. However, microscopic diseased tissue faces the same problem with [^18^F]FDG PET that it does with MRI, low tumor clarity due to high brain uptake. Additionally, gliomas identified using [^18^F]FDG have tended to be smaller than those discovered with MRI. The amino acid tracers [^11^C]MET, [^18^F]FET, and [^18^F]FDOPA have high tumor-to-background ratios compared to [^18^F]FDG. The expression of L-type amino acid transporter 1 (LAT1) and L-type amino acid transporter 2 (LAT2) in tumor cells allows the amino acids to be uptaken regardless of BBB permeability, allowing for the visualization of non-contrast-enhancing tumors [4]. However, BBB inflammation can lead to unclear imaging and delineation of the tumor. These radiotracers positively affect patient survival and have high predictive value for the course of treatment.

**Figure 2:**
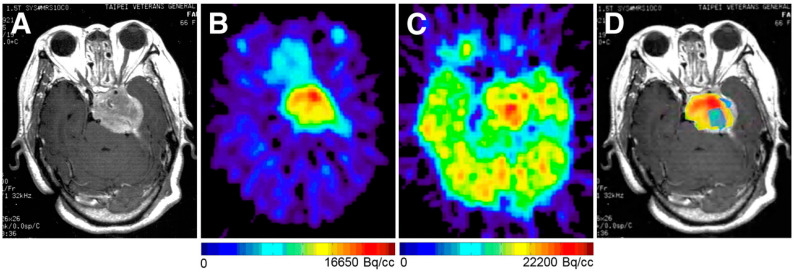
The figure demonstrates a patient with an anaplastic meningioma (Grade 3). (**A**) Contrast-enhanced MR image demonstrates a necrotic lesion in the middle fossa of the left skull base. (**B**,**C**) PET images reveal an increased uptake of both 1–11Cacetate (**B**) and 18F-FDG (**C**) in the tumor. (**D**) Co-registered PET imaging shows that the highest foci of 18F-FDG uptake (blue) coincide with areas of low 11C-acetate activity (yellow), with no overlay observed on the 11C-acetate hotspot (red). Adapted from Liu et al., 2010 [26].

A study by Liu et al. showed that [^18^F]FDG PET resulted in hypermetabolism in three meningiomas and hypometabolism in seventeen meningiomas. All 20 meningiomas had a high uptake of [^11^C]acetate. This enabled clinicians to differentiate a clearer border of the lesion than by the [^18^F]FDG information. The Standardized Uptake Value (SUV) between radiotracers varied little [26]. Unlike [^11^C]acetate, [^18^F]FDG was capable of differentiating between meningioma grades. Post-GKRS, [^11^C]acetate was more capable of monitoring tumor metabolic response than [^18^F]FDG. The two radiotracers provide complementary information to one another. [^18^F]FDG is capable of distinguishing between malignant and benign meningiomas more accurately than [^11^C]acetate, but [^11^C]acetate is capable of differentiating tumor extent (Figure 2) [26].

[^68^Ga]Ga-DOTATOC shows promise in target and organ-at-risk delineation for radiosurgery. [^68^Ga]Ga-DOTATOC PET/CT was used by Barone et al. to assess the change in SUV prior to and post-Gamma Knife radiosurgery. In this study, the standard uptake value for 58% of patients was reduced [25]. [^68^Ga]Ga-DOTATOC PET/CT is a viable diagnostic tool for meningioma diagnosis and post-operative monitoring. Other SSTR-2-based PET modalities, such as [^68^Ga]Ga-DOTATATE and DOTANOC have higher uptake in meningiomas than other metastases due to SSTR-2 receptor expression in these lesions.

Amino acid analog PET is useful for monitoring the biological response to treatment. Of these radiotracers, [^18^F]FET is the most promising. It can provide dynamic data that can allow for the derivation of a time–activity curve of [^18^F]FET uptake. Unlike [^11^C]MET and [^18^F]FDOPA, [^18^F]FET does not experience metabolic degradation or protein incorporation. [^18^F]FET PET has a diagnostic accuracy of 85% for glioblastoma patients in the differentiation of pseudoprogression from effective radiochemotherapy [15]. The use of [^18^F]FDG is limited by high glucose metabolism in brain parenchyma, which decreases tumor delineation. Furthermore, [^18^F]FDG has a high uptake in inflammatory cells, which may be caused by a variety of other diseases. Hirano et al. found that [^18^F]FDG PET was not sensitive enough to detect a malignant lymphoma [30].

Using [^18^F]FET improves the imaging of the tumor extent of cerebral neoplasms compared to MR imaging. [^18^F]FET accumulates in areas of reactive astrogliosis. This is common in the vicinity of cerebral gliomas and is a method of the CNS maintaining homeostasis under stress [28]. All rats with increased [^18^F]FET uptake had large F98-gliomas, regardless of being irradiated or non-irradiated. All tumors with low [^18^F]FET uptake showed a pronounced reactive astrogliosis. In thirty-one of thirty-three rats, the histologically determined tumor extent was congruent with the area defined by [^18^F]FET uptake. High [^18^F]FET uptake in two highly irradiated rats (150 Gy) led to the overestimation of tumor size. Reactive astrogliosis overall does not impact the [^18^F]FET uptake-determined definition of gliomas significantly.

Hypoxic brain tumors must be monitored to predict lesion aggressiveness and understand treatment effectiveness. A study by Shibahara et al. analyzed the capabilities of [^18^F]FRP-170 as a hypoxia imaging agent. [^18^F]FRP-170 has faster clearance and higher image contrast compared to previous hypoxia-measuring PET modalities [27]. In this study, [^18^F]FRP-170 PET demonstrated significant uptake with the upregulation of HIF-1alpha in glioblastomas, which was used to immunostain tumor biopsies. [^18^F]FDG PET/MR showed some correlation with the [^18^F]FRP-170 PET data, although no correlation was discovered between the [^18^F]FRP-170 PET data and the [^11^C]MET PET data. Mapelli et al. found that information obtained by [^18^F]FAZA PET had a positive correlation with Carbonic Anhydrase IX (CA-IX) and CD31 in high-grade gliomas [27]. These new methods can effectively detect and monitor hypoxic lesions pre-operatively [29].

### 4.3. Differentiating Between Lesion Recurrence and Radiation Necrosis

Differentiating between tumor recurrence and the injury of surrounding tissues due to GKRS is highly relevant to post-operative care for SRS patients. GKRS stimulates severe and chronic alterations of glucose metabolism in localized regions, causing the necrosis of surrounding healthy tissue. When radionecrotic tissue is misidentified as a recurring lesion during imaging, unnecessary and potentially harmful further treatments may occur. True radiation necrosis typically appears during a median of 7 to 11 months post-SRS [18]. This necrosis may result in cognitive impairment. Although Alongi et al. found normal cognition in patients who underwent GKRS for gliomas [36], Burzynski et al. found that the use of GKRS during the treatment of a gliosarcoma resulted in long-term cognitive deficits [38].

Typically, there is a 3:1 ratio of necrosis to recurrence post-GKRS, though both usually exist together [45]. Co-registered CT improved the visualization of anatomical structures that were obscured on MR imaging. PET increased the visualization of lesions with high metabolic activity [46]. This enabled the differentiation of stable lesions from those that were progressing. This technique allows clinicians to directly use anatomical and metabolic information, as well as time-based changes during the planning of GKRS. This clarified ambiguities during stereotactic imaging and reduced radiation exposure to non-cancerous tissues, reducing the potential for radiation necrosis. Tamura et al. found that CD133-positive gliomas survived high-dose radiation treatment, resulting in recurrence [39].

The increased uptake of [^18^F]FDG is interpreted as an increase in metabolic rate, designating the appearance of a local tumor recurrence. These recurrences were located using [18F]FDG PET in seven of the one hundred and seventy-two patients (4.1%) [34]. [^18^F]FDG PET/CT detected hypermetabolic recurrent lesions in a patient post-GKRS [50]. Surgical resection revealed radiation necrosis, not tumor tissue. What was initially understood to be a radioresistant histology was later determined to be eradicable with ablative dose regimens. SUV measurements using [^18^F]FDG PET allow for the tracking of responses to stereotactic radiosurgery, where legion progression is discovered. The relative SUV of a lesion post-GKS was linked to future progression. Those with an SUV less than 1.75 were likely to have a significant response to GKRS. However, SUV exceeding 2.75 denotes lesions that will continue to grow post-treatment [5]. This link can be used to identify patients likely to benefit from additional GKR. None of the other pre-treatment demographic or clinical characteristics were associated with response to therapy, individually or as a group.

Thirty-three weeks post-GKRS, all patients in a study conducted by Belohlávek et al. underwent both PET and MRI. There was a significantly larger difference between the survival rates of the negative and positive subgroups for [^18^F]FDG PET than the significant difference for MRI. MRI had a sensitivity of 100%, specificity of 65.3%, and an accuracy of 70.2%. [^18^F]FDG PET had a sensitivity of 75%, specificity of 93.9%, and an accuracy of 91.2% [21]. [^18^F]FDG is specific but insensitive, whereas MRI is sensitive but nonspecific. [^18^F]FDG PET offered no information to the MRI-negative group but did bring value to the positive MRI group.

Up to 26% of SRS patients have radiation necrosis from the large volume of radiation on healthy brain tissue. These damaged cells appear to be tumor recurrence on MRI images, which can confuse prognosis. [^18^F]FDG PET has an accuracy rate of 79% to 96% in differentiating between the cellular changes, yet it relies on highly proliferating cancer cells to take up the radiotracer because of the increased rate of glycolysis [7]. Neither Magnetic Resonance Spectroscopy (MRS) nor PET/CT has complete accuracy when compared to the histopathological results of the experiment, and as such cannot be considered an adequate modality for this differentiation.

MRI poorly differentiates between tumor cells and necrotic tissue, especially in cases of focal recurrence. Patients who received treatment with [^11^C]MET PET had a greater survival rate. Overall, the patients who received treatment guided by PET had a median survival rate of 18.1 months, while patients whose treatment was guided by MRI had a median survival period of 8.6 months. Patients who received PET-guided treatment had an overall lower radiation dose than those who did not. This is linked to the high accuracy of tumor delineation of this modality [19]. While the histological examination of tissues is the prominent method of differentiating between necrosis and recurrence, a noninvasive method is necessary. [^11^C]MET PET is widely regarded as both sensitive and specific. A study performed by Tomura et al. showed that [^11^C]MET PET may be preferable to [^18^F]FDG PET, apparent diffusion coefficient, and MR permeability imaging for distinguishing lesion recurrence from radiation necrosis after GKRS. [^11^C]MET typically accumulates in recurrent lesions due to the high prevalence of amino acid transporters in anaplastic tissue. This accumulation is due to passive diffusion through the damaged blood–brain barrier [20].

In a study conducted by Kits et al., [^11^C]MET PET had a high diagnostic accuracy for differentiating between lesion recurrence and radiation necrosis. [^11^C]MET PET had an accuracy of 100% for tumor recurrence and a specificity of 76% in the SUR contralateral mirror region. In the contralateral frontal cortex, [^11^C]MET PET had a sensitivity of 90% and a specificity of 78% [34]. [^11^C]MET PET can contribute to the planning of treatment, including the decision for re-GKR. However, this information does not have a high enough specificity for the actual planning of GKRS [33]. When attempting to determine if [^11^C]MET PET tumor delineation followed a Gaussian model, Simaeys et al. found that in 22 (88.0%) of the tumors, there was a match between the margins identified by MRI and those identified by [^11^C]MET PET, as well as confirmation of a 3D Gaussian model [24]. Lim et al. found that the distortion of [^11^C]MET PET imaging had a mean value of 3%. Furthermore, the PET-determined metabolic tumor volume (MTV) and MR-enhancing volume had a median matching percentage of 39.9% with PET/CT fusion and 36.8% with PET/MR fusion [32]. In fact, Lim et al. stated that this specificity would likely decrease in a clinical setting. The limitations of both PET/MR and PET/CT imaging are primarily due to low spatial resolution and accuracy, an important consideration during use in clinical settings. [^11^C]MET PET uptake was found to have a positive correlation with tumor proliferative capacity and radionecrosis [37].

The tumor tissue to normal tissue (T/N) ratio on [^11^C]MET PET/CT is affected by many factors. Jung et al. analyzed the effect of using T/N ratio cutoff values based on corrected metabolic tumor volume (MTV) on the effectiveness of [^11^C]MET PET/CT in diagnosing tumor recurrence [35]. Fifty-one of the seventy-seven lesions in the study were diagnosed as recurrent. The average T/N of recurrent lesions was 2.25, and the average T/N of necrotic lesions was 1.44. The optimal non-corrected T/N ratio was found to be 1.61, with a sensitivity of 70.6%, a specificity of 80.8%, and a diagnostic accuracy of 74.0%. The researchers determined that the optimal cutoff values for T/N groups were 1.23 for an MTV less than or equal to 0.5 cm^3^, 1.54 for an MTV between 0.5 cm^3^ and 4.0 cm^3^ or equal to 4.0 cm^3^, and 1.85 for an MTV greater than 4.0 cm^3^.

Clearly, PET imaging is more capable of detecting locally recurrent tissue than conventional imaging modalities. This is due to the difference in the metabolism of glucose and amino acid-derived radiotracers among necrotic tissues compared to recurrent lesions. However, the anatomical differences between these types of tissue are less apparent when viewed through structural scans. This demonstrates the multitude of applications PET imaging possesses, both pre- and post-operatively.

### 4.4. Combination Imaging

**Figure 3:**
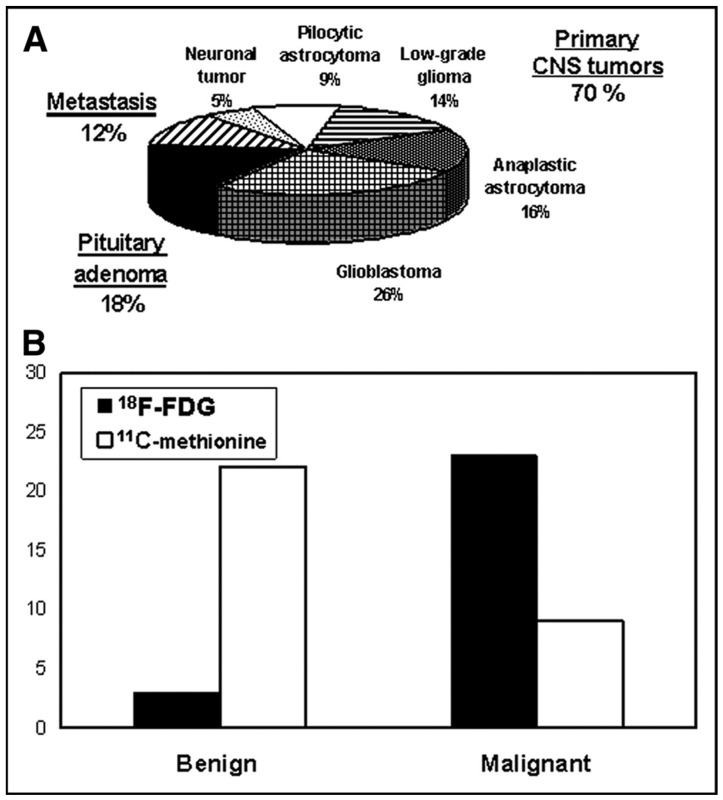
(**A**) Clinical breakdown of PET utilization for dosimetric planning in radiosurgery. (**B**) Comparative frequency of ^18^FFDG vs. ^11^C-methionine use in imaging low-grade and high-grade brain tumors. Adapted from Levivier et al., 2004 [11].

Combination imaging is a common modality used by clinicians while planning for radiosurgery. It utilizes the metabolic data obtained from PET images with the anatomical information acquired from traditional modalities such as MRI and CT. Levivier et al. found that 86% of patients had abnormal PET uptake ([^18^F]FDG or [^11^C]MET), and in 69% of patients, this information differed from the MRI-defined tumor information (Figure 3) [11].

Stefano et al. used PET/CT/MRI to assist in management through metabolism instead of histology. This modality had a sensitivity of 64.7% with a specificity of 100% [45]. PET/CT was introduced to address PET’s limited spatial resolution. However, this strategy proved insufficient, so MR imaging was subsequently co-registered with PET/CT. [^18^F]FDG PET was found to be effective in monitoring post-GKRS lesions due to lesion expectations being based on metabolic activity. However, tumor volume is difficult to determine with PET modalities due to low spatial resolution and high noise levels. An automatic biological target volume algorithm showed success in clinical settings with high accuracy and real-time performance [43].

In a study conducted by Klingenstein et al., [^68^Ga]Ga-DOTATATE PET/CT had a 100% rate of tumor specificity when detecting optic pathway tumors [35]. However, this could only be verified histopathologically in three cases. Regardless, [^68^Ga]Ga-DOTATATE PET/CT is an extremely useful tool that positively influenced the course of therapy for all patients involved [42].

Importantly, Hayenga et al. reported that [^18^F]FDG PET/CT failed to detect extracranial hemangiopericytoma lesions [47].

The majority (95%) of patients with epidural spinal metastases present with back pain.

A total of 78% of patients treated with Stereotactic Ablative Radiotherapy (SABR) had a reduction in pain scores when SUV was calculated using PET/CT for treatment planning. A decrease in SUV was directly linked to a decrease in a patient’s pain [40]. This association between pre-operative and post-operative pain levels has proved PET/CT to be useful as a supplementary treatment for palliative care in patients with spinal metastases.

Biological target volume can be segmented with [^11^C]MET PET, and Gross Tumor Volume (GTV) can be segmented with co-registered MR images. This improves the information that can be used for the planning of GKRS. Where GTV and biological tumor volume (BTV) do not match provides metabolic information about a lesion. Rundo et al. proposed an automatic multimodal PET/MRI segmentation method for GKRS [44]. This modality aims to use the high spatial resolution of MRI imaging with the high contrast of PET imaging. This method can help clinicians define a clinical target volume that incorporates multiple types of information for accurate tumor delineation.

**Figure 4:**
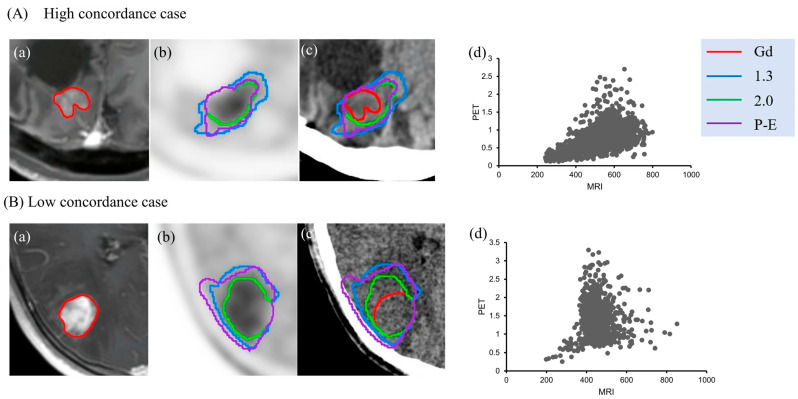
Examples of high and low concordance between Gd-enhanced and 11C-MET accumulation regions. (**A**) A 51-year-old man with a Grade 3 tumor. (**B**) A 36-year-old woman with a Grade 4 tumor. (**a**) Gd-enhanced MRI (red: CTV (Gd)). (**b**) 11C-MET PET (blue: CTV (T/N 1.3), green: CTV (T/N 2.0), purple: CTV (P-E)). (**c**) CTV contour overlaid on treatment-planning CT. (**d**) Correlation coefficient for pixel values in MRI and PET. Adapted from Niitsu et al., 2024 [56].

[^11^C]MET PET can be used in combination with MRI; however, the differences in imaging protocols make this information difficult to apply. MRI-deformed [^11^C]MET PET imaging had a large overlap with MRI, easing the use of this information for radiosurgery planning. No statistically significant difference was found between [^11^C]MET PET/MR (61.1% match rate) and MRI-deformed MET PET (63.4% match rate) modalities [41]. MRI-deformed [^11^C]MET PET showed promise for the planning of GKRS, with the ability to use functional information due to the lack of significant change in tumor volume between modalities. In a study conducted by Niitsu et al., the researchers found that the clinical target volume (CTV) defined using ^11^C-MET PET was larger than that based on the gadolinium (Gd) contrast-enhanced area, with an estimated overlap of approximately 70% at best (Figure 4) [56].

The use of Contrast-Enhanced Magnetic Resonance Imaging (CE-MRI) with static [^18^F]FET PET aids in differentiation between brain metastasis recurrence and radiation injury. These modalities have an 82% and 83% diagnostic accuracy, respectively. This increases to 90% accuracy when combined to identify the textural features of brain metastases [18]. Furthermore, this method is accessible as it requires no additional image acquisition and can be utilized by physicians with prior, standard CE-MRI, and [^18^F]FET PET images.

[^18^F]FDOPA is capable of staging and restaging brain tumors using the increased transport of the amino acid analog through the L-type large neutral amino acid transport system.

[^18^F]FDOPA PET/CT combination imaging effectively detects tumor progression and recurrence. [^18^F]FDOPA PET/CT can be used to determine tumor prognosis, to categorize a tumor, and to assess the effectiveness of treatment [48].

### 4.5. Microscopic Infiltrations

Correctly detecting and eliminating the microscopic extensions and infiltrations of a tumor is critical in preventing tumor recurrence. For this reason, identifying the optimal radiotracer and imaging modality is of utmost importance for patient outcomes. Microscopic diseased tissue faces the same problem with [^18^F]FDG PET that it does with MRI, low tumor clarity due to high brain uptake [4].

Umana et al. detected tumors missed on typical imaging modalities, known as “incidentalomas,” using [^68^Ga]Ga-DOTATOC PET/CT. [^68^Ga]Ga-DOTATOC PET/CT scans are able to detect multiple tumor regions that can receive treatment with Gamma Knife radiosurgery but are undetectable using MRI or CT [23]. The microscopic extension of lesions is often missed by MRI and CT. The suggested radiotracer makes use of markers with a high affinity for SSTR-2, and is therefore highly effective in tumors such as carcinoids and meningiomas, which have a high expression of SSTR-2. This radiotracer is especially useful for patients with tumors who are not eligible for surgery and require imaging alone to plan radiosurgery. [^18^F]FDG PET lacks sensitivity for tumors in particular regions, such as carcinoids, an area where [^68^Ga]Ga-DOTATOC PET/CT scans are particularly strong due to their high specificity in microscopic tissue detection.

Levivier et al. incorporated fiducial marker imaging to use PET data in association with GammaPlan. Researchers used a phantom to confirm that PET data allow for high spatial accuracy [6]. Furthermore, PET/MR imaging enabled the definition of target volume in gliomas. This is especially beneficial in cases with poorly defined lesions and microscopic infiltrations.

This modality improves radiosurgical outcomes, as well as enables the improved monitoring of lesion metabolism post-surgery.

### 4.6. Prognosis

PET significantly influenced prognosis and treatment outcomes in patients undergoing GKRS for brain tumors. Amino acid tracers, such as MET, FET, FPIA, and FDOPE, demonstrated a high predictive value for patient survival through tumor delineation [51,52,53]. These tracers, due to their high tumor-to-background ratios, enhance the identification of non-contrast-enhancing tumors, directly influencing patient survival and treatment outcomes [4]. In a study by Momose et al., patients treated with MET PET guidance experienced a median survival rate of 18.1 months compared to 8.6 months for those treated based on MRI alone. This highlights the improved accuracy of tumor delineation through PET imaging, which also led to lower radiation doses during treatment [11].

A study by Pirotte et al., involving 400 pediatric patients, illustrated that [^18^F]FDG and [^11^C]-Methionine PET improved surgical outcomes by enhancing tumor delineation and achieving total resection in 75% of cases. Of the first 22 patients operated on in the study, [^11^C]MET PET provided better tumor resection in 20 of them compared to MRI alone. Throughout the entire study, metabolic data influenced the surgical management of around 30% of all pediatric brain tumors. The ability of PET to provide prognostic information and differentiate between indolent and evolving tumors was crucial, particularly for cases with poorly defined MRI characteristics [12].

Other studies demonstrate PET’s ability to guide decisions on additional GKRS through the monitoring of tumor progression [5,21]. Overall, these findings underscore the pivotal role of PET in optimizing GKRS treatment strategies, enhancing diagnostic accuracy, and improving patient prognoses across various tumor types.

## 5. Conclusions

The current literature indicates that PET can serve as a primary imaging modality for the planning of GKRS, as well as for post-operative surveillance. Although performance varies by radiotracer, PET has demonstrated utility in identifying recurrent lesions post-GKRS, whereas other modalities may misinterpret recurrence as radiation necrosis. Combination imaging, such as PET/CT or PET/MRI, offers superior lesion delineation compared to single-modality imaging due to the integration of both metabolic and anatomical information. Clinically, PET should be considered in cases where conventional MRI or CT is equivocal, particularly for distinguishing recurrence from treatment-related changes. While amino acid-based tracers such as FET and MET show promise in this context, and FDG remains widely available, the current literature remains inconclusive regarding the optimal radiotracers for intracranial neoplasm definition, recurrence detection, and necrosis differentiation. We recommend the integration of PET, preferably PET/MRI where accessible, into GKRS planning and follow-up for patients with ambiguous imaging findings or complex lesions. While no formal bias tool was applied, limitations related to retrospective and small-sample studies are acknowledged. Moreover, the absence of a formal risk of bias assessment or evidence grading (e.g., ROBINS-I, GRADE) limits our ability to weigh the quality of included studies and derive strength-of-evidence conclusions. Although the majority of the available studies emphasize tumor delineation and radiosurgical planning, these elements are integral to optimizing treatment by improving targeting accuracy, minimizing radiation exposure to healthy tissue, and enhancing clinical outcomes. While the contemporary literature conveys a positive clinical opinion for the use of PET imaging in conjunction with GKRS, further research using standardized methodologies and better meta-analyses is necessary to definitively determine the optimal radiotracers for a given surgical case.

## Figures and Tables

**Table 1:** Overview of studies exploring PET imaging in the context of GKRS.

Article	Demographics/Subject Information	Utility of PET (Yes/No)	Utility of PET-MRI (Yes/No)	Radiotracers
Levivier (2002) [22]	Review of experience with 150 stereotactic biopsies, 43 neuronavigation procedures, and 34 cases treated with GKS	Yes	Yes	FDG, [^11^C]MET
Levivier (2000) [6]	Validation of technique for fiducial marker imaging, importation, and handling of PET data with GammaPlan planning software	Yes	Yes	FDG, MET
Simonova (2016) [13]	25 patients with pilocytic astrocytomas that underwent GKS	Not Applicable (N/A)	N/A	N/A
Domeki (2020) [17]	8 patients treated for esophageal cancer that developed brain metastases	N/A	N/A	N/A
Sinclair (2016) [8]	Case study	N/A	N/A	N/A
Yomo (2019) [9]	219 patients with 252 symptomatic midsize brain metastases	N/A	N/A	N/A
Castellano (2021) [4]	Review of the current literature	Yes	Yes	FDG, MET, FET, F-DOPA
Umana (2022) [23]	61 patients (18 patients with 25 brain lesions, 43 patients with 85 body lesions)	Yes	No	^(68)^Ga-DOTATOC
Galldiks (2016) [15]	Review of the current literature	Yes	No	FET, MET, F-DOPA
Tang (2008) [24]	3D Gaussian model with 25 brain metastases as experimental data	Yes	Yes	MET
Azar (2021) [10]	Review of the current literature	Yes	Yes	FDG, MET
Momose (2014) [19]	88 patients that underwent GK-SRS	Yes	Yes	MET
Barone (2021) [25]	20 patients with pre-operative PET/CT	Yes	No	^(68)^Ga-DOTATOC
Liu (2010) [26]	22 meningioma patients	Yes	No	1-(11)C-acetate, ^(18)^F-FDG
Shibahara (2010) [27]	8 patients with FRP-170 injections and PET imaging	Yes	Yes	FRP-170, FDG
Piroth (2013) [28]	33 Fisher CDF rats with F98-glioma cell implants	Yes	No	^(18)^F-FET
Mapelli (2021) [29]	20 patients with brain MRI suggestive of high-grade glioma	Yes	No	^18^F-FAZA
Hirano (2011) [30]	Case report	Yes	No	FDG
Schindler (2006) [31]	Case report	Yes	No	FDG
Travers (2021) [7]	242 patients with previous whole-brain radiation therapy or sterotactic radiosurgery	No	No	^(18)^F-FDG
Leiva-Salinas (2019) [5]	85 patients with brain metastases from non-CNS neoplasms treated with GKS	Yes	Yes	FDG
Lim (2019) [32]	Imaging from 14 metastatic brain tumors	No	Yes	MET
Belohlávek (2003) [21]	25 patients with 57 metastases treated with stereotactic radiosurgery	Yes	No	FDG
Tomura (2017) [20]	15 GKS patients with metastatic brain tumors	Yes	No	MET, FDG
Kits (2018) [33]	30 patients with irradiated intracranial tumors	Yes	No	MET
Lippitz (2004) [34]	215 GKS patients	Yes	No	FDG
Jung (2017) [35]	48 patients with 77 metastatic brain lesions	Yes	No	MET
Alongi (2018) [36]	6 patients with possible late brain effects following radiation therapy	Yes	No	^18^F-FDG
Szeifert (2002) [37]	Case report	Yes	No	MET
Burzynski (2014) [38]	Case report	Yes	No	Not specified
Tamura (2010) [39]	32 GKS/EBRT patients with malignant gliomas	N/A	N/A	N/A
Almeida (2018) [40]	72 patients with spinal metastases treated with stereotactic ablative radiation therapy	Yes	No	FDG
Lohmann (2018) [18]	52 patients with new or progressive contrast-enhancing brain lesions on MRI following radiotherapy	Yes	Yes	FET
Jung (2019) [41]	12 newly developed metastatic brain tumors	Yes	Yes	MET
Klingenstein (2015) [42]	13 patients with ambiguous, symptomatic lesions of the optic pathway	Yes	No	Ga-68-DOTA-TATE
Stefano (2016) [43]	Algorithm for BTV delination	No	No	N/A
Levivier (2004) [11]	57 GKS patients with stereotactic PET on the same day as stereotactic MRI or CT	Yes	Yes	FDG, MET
Rundo (2017) [44]	19 brain metastatic tumors analyzed by a novel automatic PET/MRI segmentation method for GKS patients	Yes	Yes	MET
Torrens (2016) [45]	27 PET/CT studies co-registered with MRI performed on 16 patients	Yes	Yes	FDG
Koga (2009) [46]	Co-registration from 180 radiosurgery sessions	Yes	Yes	FDG
Hayenga (2019) [47]	Case report	No	No	FDG
Marafi (2022) [48]	Case report	Yes	No	^(18)^F-DOPA
Pirotte (2007) [12]	400 consecutive pediatric brain tumors	Yes	No	FDG, MET
Levivier (2004) [49]	80 LGK patients treated with combination MR/CT and PET guidance	Yes	Yes	Not specified
Register (2010) [50]	Case report	Yes	No	FDG
Lo Greco (2022) [51]	Review of the current literature	N/A	N/A	N/A
De Marco (2022) [52]	Systematic Review	Yes	Yes	MET, FET, ^18^F-DOPA, FDG
Islam (2023) [53]	Ten adult glioma patients imaged with FPIA PET/MRI protocols	Yes	Yes	FPIA

## Abbreviations

[^11^C]acetate	1-11C-acetate
BBB	Blood–Brain Barrier
BTV	Biological Tumor Volume
CA-IX	Carbonic Anhydrase IX
CE-MRI	Contrast-Enhanced Magnetic Resonance Imaging
CNS	Central Nervous System
CT	Computed Tomography
[^68^Ga]Ga-DOTANOC	[^68^Ga]Ga-DOTA-1-NaI3-octreotide
[^68^Ga]Ga-DOTATATE	[^68^Ga]Ga-DOTA,Tyr3]-octreotate
[^68^Ga]Ga-DOTATOC	[^68^Ga]Ga-DOTA-D-Phe-Tyr3-octreotide
[^18^F]FAZA	[^18^F]fluoroazomycin arabinoside
[^18^F]FET	[^18^F]fluoro-ethyl-tyrosine
[^18^F]FDG	[^18^F]fluorodeoxyglucose
[^18^F]FDOPA	[^18^F]FDOPA-6-[^18^F]fluoro-l-dopa
[^18^F]FRP-170 FPIA	[^18^F]fluoro-methyl-2-nitroimidazole [^18^F]fluoropivalate
GKRS	Gamma Knife Radiosurgery
GLUT-1	Glucose Transporter 1
GTV	Gross Tumor Volume
HIF-1alpha	Hypoxia-Inducible Factor 1-alpha
LAT1	L-type amino acid transporter 1
LAT2	L-type amino acid transporter 2
[^11^C]MET	[^11^C]methionine
MRI	Magnetic Resonance Imaging
MRS	Magnetic Resonance Spectroscopy
MTV	Metabolic Tumor Volume
PET	Positron Emission Tomography
PRISMA	Preferred Reporting Items for Systematic Reviews and Meta-Analyses
RCTs	Randomized Controlled Trials
SABR	Stereotactic Ablative Radiotherapy
SRS	Stereotactic Radiosurgery
SSTR	Somatostatin Receptor
SSTR-2	Somatostatin Receptor Subtype 2
SUV	Standardized Uptake Value
T/N	Tumor Tissue to Normal Tissue Ratio

## References

[1] Desai R., Rich K.M. (2020). Therapeutic Role of Gamma Knife Stereotactic Radiosurgery in Neuro-Oncology. Mo. Med..

[2] Jones T. (1996). The role of positron emission tomography within the spectrum of medical imaging. Eur. J. Nucl. Med..

[3] Jones T., Townsend D. (2017). History and future technical innovation in positron emission tomography. J. Med. Imaging.

[4] Castellano A., Bailo M., Cicone F., Carideo L., Quartuccio N., Mortini P., Falini A., Cascini G.L., Minniti G. (2021). Advanced Imaging Techniques for Radiotherapy Planning of Gliomas. Cancers.

[5] Leiva-Salinas C., Muttikkal T.J.E., Flors L., Puig J., Wintermark M., Patrie J.T., Rehm P.K., Sheehan J.P., Schiff D. (2019). FDG PET/MRI Coregistration Helps Predict Response to Gamma Knife Radiosurgery in Patients with Brain Metastases. Am. J. Roentgenol..

[6] Levivier M., Wikier D., Goldman S., David P., Metens T., Massager N., Gerosa M., Devriendt D., Desmedt F., Simon S. (2000). Integration of the metabolic data of positron emission tomography in the dosimetry planning of radiosurgery with the gamma knife: Early experience with brain tumors. Technical note. J. Neurosurg..

[7] Travers S., Joshi K., Miller D.C., Singh A., Nada A., Biedermann G., Cousins J.P., Litofsky N.S. (2021). Reliability of Magnetic Resonance Spectroscopy and Positron Emission Tomography Computed Tomography in Differentiating Metastatic Brain Tumor Recurrence from Radiation Necrosis. World Neurosurg..

[8] Sinclair G., Bartek J., Martin H., Barsoum P., Dodoo E. (2016). Adaptive hypofractionated gamma knife radiosurgery for a large brainstem metastasis. Surg. Neurol. Int..

[9] Yomo S., Oda K., Oguchi K. (2019). Single- versus 2-session Gamma Knife surgery for symptomatic midsize brain metastases: A propensity score-matched analysis. J. Neurosurg..

[10] Azar M., Mohsenian Sisakht A., Kazemi Gazik F., Shahrokhi P., Rastegar K., Karamzade-Ziarati N. (2021). PET-guided gamma knife radiosurgery in brain tumors: A brief review. Clin. Transl. Imaging.

[11] Levivier M., Massager N., Wikler D., Lorenzoni J., Ruiz S., Devriendt D., David P., Desmedt F., Simon S., Van Houtte P. (2004). Use of stereotactic PET images in dosimetry planning of radiosurgery for brain tumors: Clinical experience and proposed classification. J. Nucl. Med..

[12] Pirotte B., Acerbi F., Lubansu A., Goldman S., Brotchi J., Levivier M. (2007). PET imaging in the surgical management of pediatric brain tumors. Child’s Nerv. Syst..

[13] Simonova G., Kozubikova P., Liscak R., Novotny J. (2016). Leksell Gamma Knife treatment for pilocytic astrocytomas: Long-term results. J. Neurosurg. Pediatr..

[14] St George E.J., Plowman P.N. (2002). The role of the Gamma Knife and PET in neuro-oncology. CME Cancer Med..

[15] Galldiks N., Law I., Pope W.B., Arbizu J., Langen K.J. (2016). The use of amino acid PET and conventional MRI for monitoring of brain tumor therapy. NeuroImage Clin..

[16] Yano H., Shinoda J., Iwama T. (2017). Clinical Utility of Positron Emission Tomography in Patients with Malignant Glioma. Neurol. Med.-Chir..

[17] Domeki Y., Nakajima M., Takahashi M., Kikuchi M., Yokoyama H., Ogata H., Okamoto K., Yamaguchi S., Sasaki K., Tsuchioka T. (2020). Treatment strategy for brain metastases from esophageal cancer. Tumori J..

[18] Lohmann P., Kocher M., Ceccon G., Bauer E.K., Stoffels G., Viswanathan S., Ruge M.I., Neumaier B., Shah N.J., Fink G.R. (2018). Combined FET PET/MRI radiomics differentiates radiation injury from recurrent brain metastasis. NeuroImage Clin..

[19] Momose T., Nariai T., Kawabe T., Inaji M., Tanaka Y., Watanabe S., Maehara T., Oda K., Ishii K., Ishiwata K. (2014). Clinical benefit of 11C methionine PET imaging as a planning modality for radiosurgery of previously irradiated recurrent brain metastases. Clin. Nucl. Med..

[20] Tomura N., Kokubun M., Saginoya T., Mizuno Y., Kikuchi Y. (2017). Differentiation between Treatment-Induced Necrosis and Recurrent Tumors in Patients with Metastatic Brain Tumors: Comparison among 11C-Methionine-PET, FDG-PET, MR Permeability Imaging, and MRI-ADC-Preliminary Results. Am. J. Neuroradiol..

[21] Belohlávek O., Simonová G., Kantorová I., Novotný J., Liscák R. (2003). Brain metastases after stereotactic radiosurgery using the Leksell gamma knife: Can FDG PET help to differentiate radionecrosis from tumour progression?. Eur. J. Nucl. Med. Mol Imaging.

[22] Levivier M., Wikler D., Massager N., David P., Devriendt D., Lorenzoni J., Pirotte B., Desmedt F., Simon S., Goldman S. (2002). The integration of metabolic imaging in stereotactic procedures including radiosurgery: A review. J. Neurosurg..

[23] Umana G.E., Ferini G., Harikar M.M., Venkataram T., Costanzo R., Scalia G., Palmisciano P., Brunasso L., Paolini F., Sciortino A. (2022). Detection of “Incidentalomas” on Brain and Body 68Ga-DOTATOC-PET Scans: A Retrospective Study and Case Illustration. Anticancer Res..

[24] Tang B.N., Van Simaeys G., Devriendt D., Sadeghi N., Dewitte O., Massager N., David P., Levivier M., Goldman S. (2008). Three-dimensional Gaussian model to define brain metastasis limits on 11C-methionine PET. Radiother. Oncol..

[25] Barone F., Inserra F., Scalia G., Ippolito M., Cosentino S., Crea A., Sabini M.G., Valastro L., Patti I.V., Mele S. (2021). 68Ga-DOTATOC PET/CT Follow Up after Single or Hypofractionated Gamma Knife ICON Radiosurgery for Meningioma Patients. Brain Sci..

[26] Liu R.S., Chang C.P., Guo W.Y., Pan D.H., Ho D.M., Chang C.W., Yang B.H., Wu L.C., Yeh S.H. (2010). 1-11C-acetate versus 18F-FDG PET in detection of meningioma and monitoring the effect of gamma-knife radiosurgery. J. Nucl. Med..

[27] Shibahara I., Kumabe T., Kanamori M., Saito R., Sonoda Y., Watanabe M., Iwata R., Higano S., Takanami K., Takai Y. (2010). Imaging of hypoxic lesions in patients with gliomas by using positron emission tomography with 1-(2-[18F] fluoro-1-[hydroxymethyl]ethoxy)methyl-2-nitroimidazole, a new 18F-labeled 2-nitroimidazole analog. J. Neurosurg..

[28] Piroth M.D., Prasath J., Willuweit A., Stoffels G., Sellhaus B., van Osterhout A., Geisler S., Shah N.J., Eble M.J., Coenen H.H. (2013). Uptake of O-(2-[18F]fluoroethyl)-L-tyrosine in reactive astrocytosis in the vicinity of cerebral gliomas. Nucl. Med. Biol..

[29] Mapelli P., Callea M., Fallanca F., Castellano A., Bailo M., Scifo P., Bettinardi V., Conte G.M., Monterisi C., Rancoita P.M.V. (2021). 18F-FAZA PET/CT in pretreatment assessment of hypoxic status in high-grade glioma: Correlation with hypoxia immunohistochemical biomarkers. Nucl. Med. Commun..

[30] Hirano H., Tashiro Y., Fujio S., Goto M., Arita K. (2011). Diffuse large B-cell lymphoma within a cavernous hemangioma of the cavernous sinus. Brain Tumor Pathol..

[31] Schindler K., Christ E.R., Mindermann T., Wieser H.G. (2006). Transient MR changes and symptomatic epilepsy following gamma knife treatment of a residual GH-secreting pituitary adenoma in the cavernous sinus. Acta Neurochir..

[32] Lim S.H., Jung T.Y., Jung S., Kim I.Y., Moon K.S., Kwon S.Y., Jang W.Y. (2019). Quantitative Feasibility Evaluation of 11C-Methionine Positron Emission Tomography Images in Gamma Knife Radiosurgery: Phantom-Based Study and Clinical Application. J. Korean Neurosurg. Soc..

[33] Kits A., Martin H., Sanchez-Crespo A., Delgado A.F. (2018). Diagnostic accuracy of 11C-methionine PET in detecting neuropathologically confirmed recurrent brain tumor after radiation therapy. Ann. Nucl. Med..

[34] Lippitz B.E., Kraepelien T., Hautanen K., Ritzling M., Rähn T., Ulfarsson E., Boethius J. (2004). Gamma knife radiosurgery for patients with multiple cerebral metastases. Acta Neurochir. Suppl..

[35] Jung T.Y., Kim I.Y., Lim S.H., Park K.S., Kim D.Y., Jung S., Moon K.S., Jang W.Y., Kang S.R., Cho S.G. (2017). Optimization of diagnostic performance for differentiation of recurrence from radiation necrosis in patients with metastatic brain tumors using tumor volume-corrected 11C-methionine uptake. EJNMMI Res..

[36] Alongi P., Iaccarino L., Losa M., Del Vecchio A., Gerevini S., Plebani V., Di Muzio N., Mortini P., Gianolli L., Perani D. (2018). PET Evaluation of Late Cerebral Effect in Advanced Radiation Therapy Techniques for Cranial Base Tumors. Curr. Radiopharm..

[37] Szeifert G.T., Massager N., Brotchi J., Levivier M. (2002). Morphological redifferentiation in a malignant astrocytic tumor after gamma knife radiosurgery. J. Neurosurg..

[38] Burzynski S.R., Janicki T.J., Burzynski G.S., Marszalek A. (2014). Long-term survival (>13 years) in a child with recurrent diffuse pontine gliosarcoma: A case report. J. Pediatr. Hematol. Oncol..

[39] Tamura K., Aoyagi M., Wakimoto H., Ando N., Nariai T., Yamamoto M., Ohno K. (2010). Accumulation of CD133-positive glioma cells after high-dose irradiation by Gamma Knife surgery plus external beam radiation. J. Neurosurg..

[40] Almeida N.D., Adams C., Davis G.L., Starke R.M., Buro J., Nasr N., McRae D., Cernica G., Caputy A., Hong R. (2018). Effectiveness of Positron Emission Tomography/Computed Tomography as a Guide for Palliative Radiation Therapy for Spinal Metastases. World Neurosurg..

[41] Jung T.Y., Jung S., Ryu H.S., Kim I.Y., Jang W.Y., Moon K.S., Lim S.H., Kim D.Y., Kang S.R., Min J.J. (2019). The Application of Magnetic Resonance Imaging-Deformed 11C-Methionine-Positron Emission Tomography Images in Stereotactic Radiosurgery. Stereotact. Funct. Neurosurg..

[42] Klingenstein A., Haug A.R., Miller C., Hintschich C. (2015). Ga-68-DOTA-TATE PET/CT for discrimination of tumors of the optic pathway. Orbit.

[43] Stefano A., Vitabile S., Russo G., Ippolito M., Marletta F., D’arrigo C., D’urso D., Gambino O., Pirrone R., Ardizzone E. (2016). A fully automatic method for biological target volume segmentation of brain metastases. Int. J. Imaging Syst. Technol..

[44] Rundo L., Stefano A., Militello C., Russo G., Sabini M.G., D’Arrigo C., Marletta F., Ippolito M., Mauri G., Vitabile S. (2017). A fully automatic approach for multimodal PET and MR image segmentation in gamma knife treatment planning. Comput. Methods Programs Biomed..

[45] Torrens M., Malamitsi J., Karaiskos P., Valotassiou V., Laspas F., Andreou J., Stergiou C., Prassopoulos V. (2016). Although Non-diagnostic Between Necrosis and Recurrence, FDG PET/CT Assists Management of Brain Tumours After Radiosurgery. In Vivo.

[46] Koga T., Maruyama K., Igaki H., Tago M., Saito N. (2009). The value of image co ration during stereotactic radiosurgery. Acta Neurochir..

[47] Hayenga H.N., Bishop A.J., Wardak Z., Sen C., Mickey B. (2019). Intraspinal Dissemination and Local Recurrence of an Intracranial Hemangiopericytoma. World Neurosurg..

[48] Marafi F., Sadeq A., Esmail A., Usmani S. (2022). Case of Adult Metastatic Medulloblastoma Demonstrated on 18F-DOPA PET/CT. Clin. Nucl. Med..

[49] Levivier M., Massager N., Wikler D., Goldman S. (2004). Modern multimodal neuroimaging for radiosurgery: The example of PET scan integration. Gamma Knife Radiosurgery.

[50] Register S., Clarke J.W., Chaudhury A.R., Lo S.S. (2010). Pathologic complete response of a solitary melanoma brain metastasis after local ablative radiation therapy: Case report. Med. Oncol..

[51] Lo Greco M.C., Milazzotto R., Liardo R.L.E., Acquavia G., La Rocca M., Altieri R., Certo F., Barbagallo G.M., Basile A., Foti P.V. (2022). Relapsing High-Grade Glioma from Peritumoral Zone: Critical Review of Radiotherapy Treatment Options. Brain Sci..

[52] De Marco R., Pesaresi A., Bianconi A., Zotta M., Deandreis D., Morana G., Zeppa P., Melcarne A., Garbossa D., Cofano F. (2022). A Systematic Review of Amino Acid PET Imaging in Adult-Type High-Grade Glioma Surgery: A Neurosurgeon’s Perspective. Cancers.

[53] Islam S., Inglese M., Grech-Sollars M., Aravind P., Dubash S., Barwick T.D., O’nEill K., Wang J., Saleem A., O’cAllaghan J. (2023). Feasibility of [18F]fluoropivalate hybrid PET/MRI for imaging lower and higher grade glioma: A prospective first-in-patient pilot study. Eur. J. Nucl. Med..

[54] de Zwart P.L., van Dijken B.R.J., Holtman G.A., Stormezand G.N., Dierckx R.A.J.O., Jan van Laar P., van der Hoorn A. (2016). Diagnostic Accuracy of PET Tracers for the Differentiation of Tumor Progression from Treatment-Related Changes in High-Grade Glioma: A Systematic Review and Metaanalysis. J. Nucl. Med..

[55] Jafari E., Assadi M., Nasiri M., Ahmadzadehfar H. (2025). Targets for Molecular Imaging of Neuroendocrine Tumors (NETs): An Overview and Update. Semin. Nucl. Med..

[56] Niitsu H., Fukumitsu N., Tanaka K., Mizumoto M., Nakai K., Matsuda M., Ishikawa E., Hatano K., Hashimoto T., Kamizawa S. (2024). Methyl-^11^C-L-methionine positron emission tomography for radiotherapy planning for recurrent malignant glioma. Ann. Nucl. Med..

